# Automatic intra-subject registration and fusion of multimodal cochlea 3D clinical images

**DOI:** 10.1371/journal.pone.0264449

**Published:** 2022-03-02

**Authors:** Ibraheem Al-Dhamari, Rania Helal, Olesia Morozova, Tougan Abdelaziz, Roland Jacob, Dietrich Paulus, Stephan Waldeck

**Affiliations:** 1 Computer Vision Department, Koblenz University, Koblenz, Germany; 2 Radiodiagnosis Dept., Ain Shams University, Cairo, Egypt; 3 HNO Plus, Koblenz, Germany; 4 Interventional Radiology and Neuroradiology Dept., Military Hospital Koblenz, Koblenz, Germany; University of Alberta, CANADA

## Abstract

**Background:**

The postoperative imaging assessment of Cochlear Implant (CI) patients is imperative. The main obstacle is that Magnetic Resonance imaging (MR) is contraindicated or hindered by significant artefacts in most cases with CIs. This study describes an automatic cochlear image registration and fusion method that aims to help radiologists and surgeons to process pre-and postoperative 3D multimodal imaging studies in cochlear implant (CI) patients.

**Methods and findings:**

We propose a new registration method, Automatic Cochlea Image Registration (ACIR-v3), which uses a stochastic quasi-Newton optimiser with a mutual information metric to find 3D rigid transform parameters for registration of preoperative and postoperative CI imaging. The method was tested against a clinical cochlear imaging dataset that contains 131 multimodal 3D imaging studies of 41 CI patients with preoperative and postoperative images. The preoperative images were MR, Multidetector Computed Tomography (MDCT) or Cone Beam Computed Tomography (CBCT) while the postoperative were CBCT. The average root mean squared error of ACIR-v3 method was 0.41 mm with a standard deviation of 0.39 mm. The results were evaluated quantitatively using the mean squared error of two 3D landmarks located manually by two neuroradiology experts in each image and compared to other previously known registration methods, e.g. Fast Preconditioner Stochastic Gradient Descent, in terms of accuracy and speed.

**Conclusions:**

Our method, ACIR-v3, produces high resolution images in the postoperative stage and allows for visualisation of the accurate anatomical details of the MRI with the absence of significant metallic artefacts. The method is implemented as an open-source plugin for 3D Slicer tool.

## Introduction

The cochlea is a principal part of the inner ear that plays a crucial role in hearing, filtering frequency coded auditory signals, and transmitting them to the brain. The number of patients presenting with sensorineural hearing loss has increased over the years and Cochlear Implantation (CI) is gaining popularity as a treatment option for these patients. The electrode array in CIs simulate the function of the cochlea, which in many cases allows patients to communicate with others and enjoy a normal social life.

The preoperative CI imaging examination relies on Multidetector CT (MDCT) or more recently the Cone Beam CT (CBCT) for the assessment of the bony labyrinth. The Magnetic Resonance imaging (MR) is used mainly for evaluation of the membranous labyrinth and the intracranial vestibulocochlear nerve.

However, in the postoperative period, MR is contraindicated in most cases with CIs, and even if performed, assessment is impossible due to the pronounced metallic artefact of the CI electrode within the cochlea. Therefore postoperative assessment relies entirely on computed tomographic imaging (including MDCT or CBCT) or the conventional projection radiography (X-ray) [1]. In the postoperative stage, it is important to accurately assess the position of the intracochlear electrode in the scala tympani to rule out intracochlear malpositioning. MDCT and CBCT can only visualise the bony cochlea; a direct assessment of the cochlea scalae is only possible to a limited extent. Multimodal image fusion between preoperative MR imaging and postoperative MDCT/CBCT examination can provide a solution to this problem.

**Fig 1:**
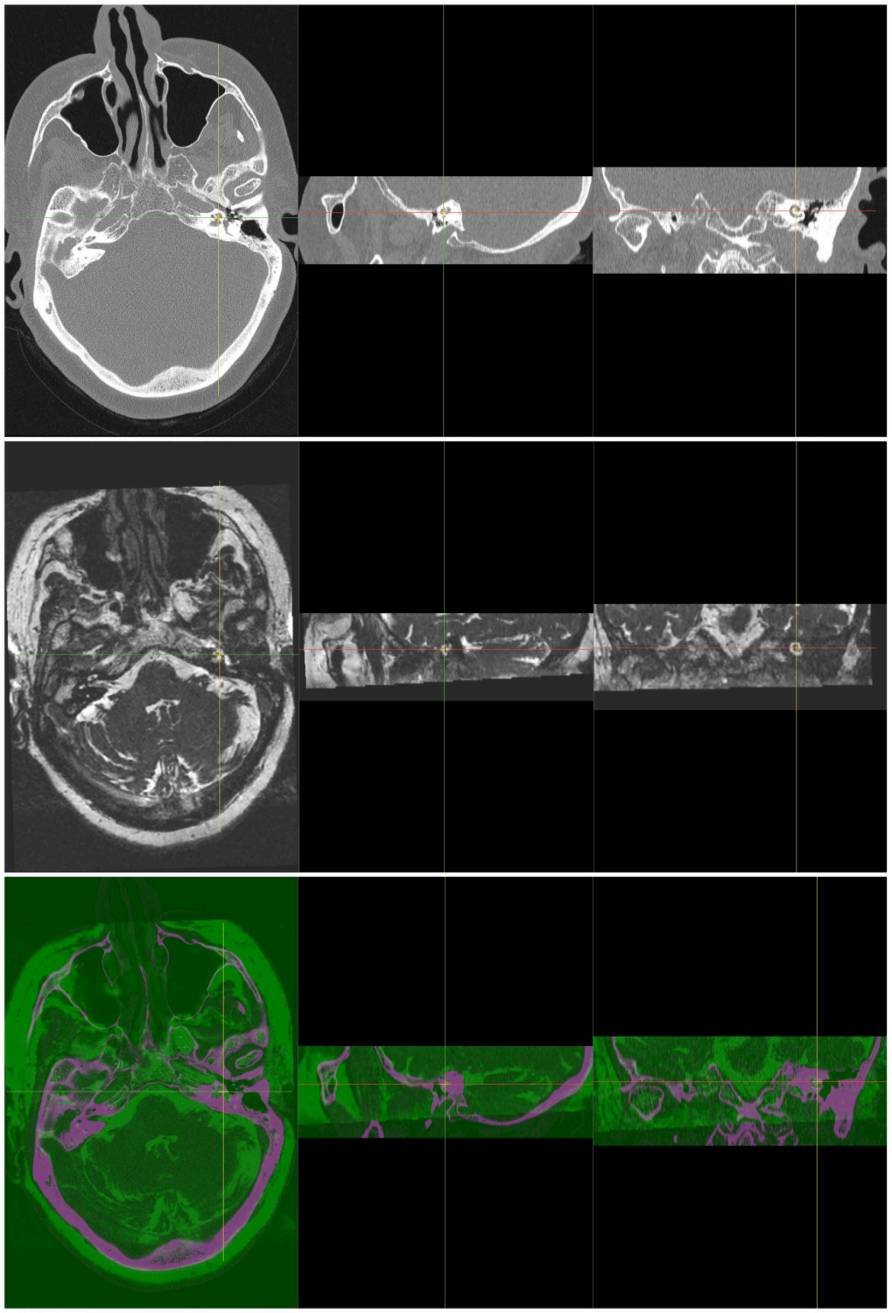
Two input 3D cochlea images of the same patient, CT as a fixed image (top) and MR as a moving image (middle). The bottom image is the registered and fused 3D image, with magenta colour representing the MR part and green colour representing the CT part. Each image has 3 views, from left to right: axial, sagittal and coronal.

Image fusion combines different images to create a single more informative image. The new fused image has features from all the input images e.g. one can see the bones and the soft tissue simultaneously from fused CT and MR images, see Fig 1. There are many methods of image fusion [2]. The method we used is a simple matrix addition which is a very fast operation that takes only a small part of a second in today’s computers e.g.:

$$\begin{array}{c} I_{fused}=I_{A}+I_{B} \end{array}$$ (1)

For an efficient fusion, images must be correctly aligned to the same physical space using image registration. Manually performing this process takes a lot of time and neuroradiological expertise. Automating this process using an image registration method not only saves a huge amount of time but can also significantly improve the repeatable quality of the results.

### Image registration

**Fig 2:**
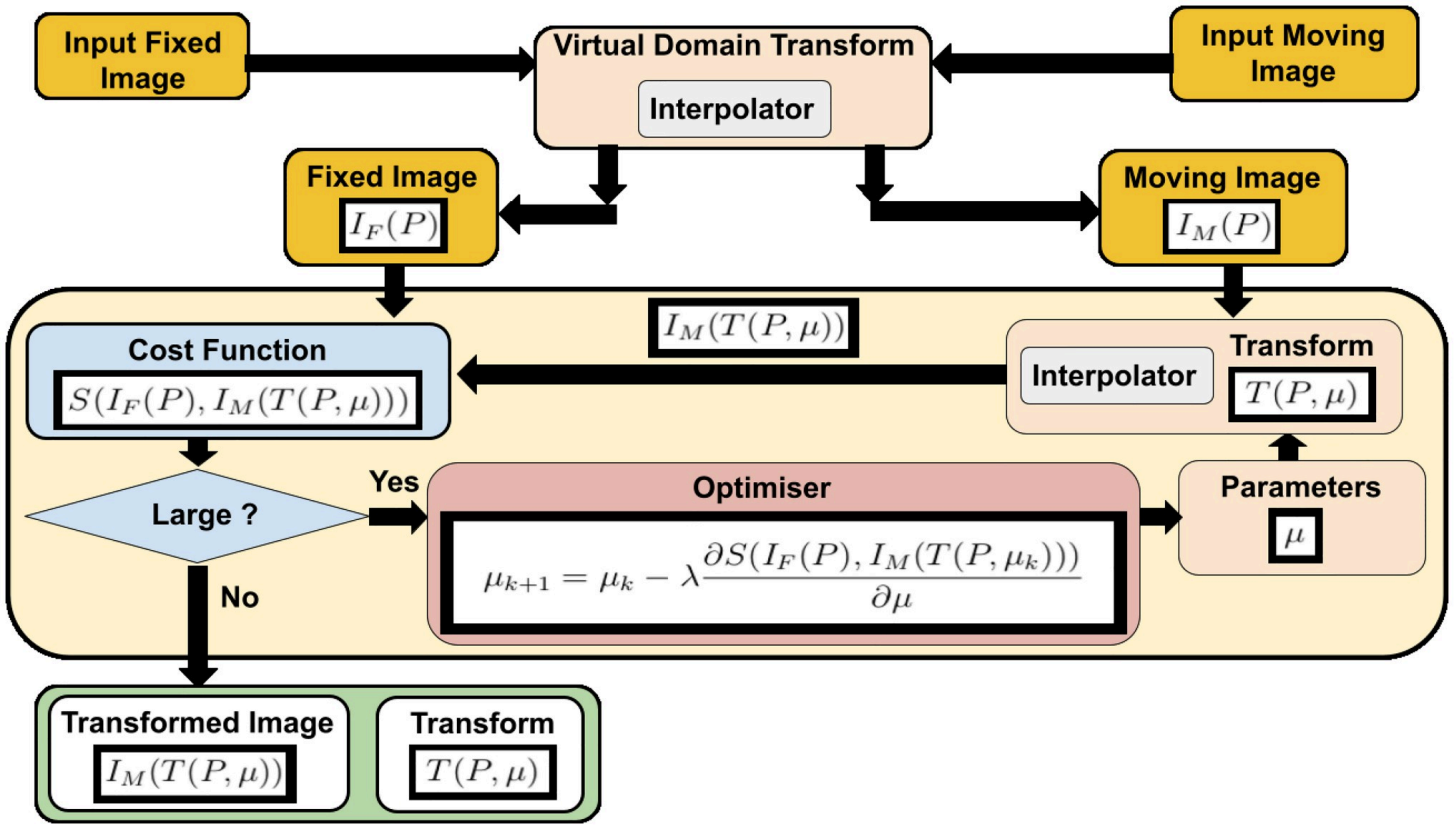
The main components of image registration: Input images, cost function, optimiser, and a transform. The virtual domain transform deals with the issue of different image resolution.

The image registration [3–5] is the problem of finding a transformation *T* that aligns one or more images to a reference image. This transformation transforms the points of an image to the same location of the points in the reference image. Intra-subject medical image registration aligns images of the same patient while inter-subject registration aligns images of different patients. Fig 2 shows the main registration components: a fixed and a moving image, a cost function, an optimiser, and a transformation. The first transformation handles the issue of different resolutions by mapping both input images to a virtual domain in the images’ physical space. This takes place before the optimisation process starts. In this implementation, medical image registration is not performed in the images’ space [6].

Mathematically, the transform is a function of the image points *P* and the transform’s parameters *μ* where it takes an image point (or a vector of points) and outputs the image transformed point (or a vector of transformed points). We can write this transform as *T*(*P*, *μ*) where *P* is the image coordinates vector. The second input to the transformation’s function *T* is *μ* which is a vector that contains the transformation’s parameters.

The transformation transforms an image called a moving image *I*_*M*_(*P*) to another image called a fixed image *I*_*F*_(*P*), where *P* is a vector of all image point coordinates. The image itself can be considered a function of points where the output is the intensity (or color) values of the point locations.

Finding the parameters of this transformation is a challenging task. Hence, the general registration problem is still unsolved and many papers are published every year trying to solve different aspects of this problem.

Optimisation techniques [7] are usually used to find these parameters. An optimiser, such as Gradient Descent (GD) [8], tries to find these parameters by minimising a cost function of the input images. Popular image registration cost functions use similarity metrics such as Mean Squared Error *S*_*MSE*_(*I*_*F*_, *I*_*M*_) [9] or Mutual Information *S*_*MI*_(*I*_*F*_, *I*_*M*_) [10]. Note that by minimising the similarity metric value, we are maximizing the similarity between the fixed and the transformed moving images.

The optimisation problem is stated as follows:

$$\begin{array}{c} \tilde{\mu }=arg\;min\;S\left(I_{F}\left(P\right),I_{M}\left(T\left(P,\mu \right)\right)\right), \end{array}$$ (2)

where *μ* is the transformation’s parameters’ vector, *I*_*F*_ and *I*_*M*_ are the fixed and the moving images, *P* is the points’ coordinate vector, *S* is the similarity metric function, and *T*(*P*, *μ*) is the transformation’s function.

In the GD optimiser, the new parameters are updated using the derivative of the cost function with respect to the transformation’s parameters, e.g.:

$$\begin{array}{c} \mu_{k+1}=\mu_{k}-\lambda \frac{\partial S(I_{F}\left(P\right),I_{M}\left(T\left(P,\mu_{k}\right)\right))}{\partial \mu }\;, \end{array}$$ (3)

where *k* is the optimisation iteration, λ represents the step-size or the learning rate of the GD. The derivative of the similarity metric with respect to the transformation’s parameters is required for Eq (3). Using the chain rule, this derivative can be divided into two main parts:

$$\begin{array}{c} \frac{\partial S}{\partial \mu }=\sum_{j=0}^{n-1}(\frac{\partial S(I_{F}\left(p_{j}\right),I_{M}\left(T\left(p_{j},\mu_{k}\right)\right))}{\partial T(p_{j})}\;\frac{\partial T(p_{j},\mu_{k})}{\partial \mu })\;, \end{array}$$ (4)

where $\frac{\partial S}{\partial \mu }$ is the first order derivative of the similarity metric *S* with respect to the transformation’s parameters *μ* at *k* optimisation’s iteration, *p*_*j*_ is an image pixel coordinate, and *n* is the total number of the image’s pixels. The first part of the right-hand side in Eq (4) requires the derivative of the similarity metric and the transformed moving image. The second part $\frac{\partial T(p_{j},\mu_{k})}{\partial \mu }$ is called *J*_*μ*_ and it is similar to the Jacobian matrix of the transformation as it represents the transformation’s partial derivatives at each image point with respect to the transformation’s parameters. The term *J*_*μ*_ is computed at each optimisation iteration. For a large image size and complex transformation, the computation requires a lot of time to complete. To solve this issue, the Stochastic Gradient Descent (SGD) optimisation approach [11, 12] uses samples instead of the whole image. Sometimes these samples are taken randomly. The stochastic approach allows fewer computations and provides better results in practice. The parameters updating rule of SGD is:

$$\begin{array}{c} \mu_{k+1}=\mu_{k}-\lambda \frac{\partial S({\hat{I}}_{F}\left(P\right),{\hat{I}}_{M}\left(T\left(P,\mu_{k}\right)\right))}{\partial \mu }\;, \end{array}$$ (5)

where ${\hat{I}}_{F}$ and ${\hat{I}}_{M}$ represent samples from the input images *I*_*F*_ and *I*_*M*_.

### Aim of the work

The aim of this work is to propose and evaluate a new relatively easy and fast automatic cochlear image registration and fusion method utilizing simple computer hardware and software to align and fuse pre-and postoperative multimodal cochlear images in cochlear implant (CI) patients.

## Materials and methods

### Ethics statement

The use of all human datasets in our experiments were approved by the Ethics Committee of the Rheinland-Pfalz state, Germany (Landesaerztekammer Rheinland-Pfalz, Koerperschaft des oeffentlichen Rechts) with request number 2021–15895-retrospektiv.

We conducted a retrospective review from 2014 to 2020 using our institution’s CI database to retrieve the preoperative and postoperative imaging data of patients who underwent CI surgery. Exclusion criteria were the presence of congenital cochlear anomalies or severe artifacts distorting the image quality.

### Dataset

The study included 41 CI patients of different genders and ages with a total of about 131 preoperative and postoperative imaging studies from different modalities (MR, MDCT and CBCT). All the patients’ information was anonymised.

All preoperative MR images were done on a Siemens Avanto 1.5 Tesla device. The sequence used was transverse T2-spc 3D sequence with the following specifications: Repetition time (TR) 9.1 ms Echo time (TE) 3.62 ms, Flip angle 80 degrees; pixel bandwidth 130Hz/Px; thickness 0.8 mm; voxel size 0.7x0.5x;0.8 mm; FOV selection 200 mm; Matrix size 384x384; SNR 1.0; Acquisition time 3:30 min. Each used image has a size of 384 × 512 × 64 voxels with 0.39x0.39x × 0.7 mm spacing.

All preoperative MDCT images were done on a Siemens Sensation Cardiac 64 Slice. Each image was taken with these parameters: KV = 120; mAS = 140; matrix = 512x512; FOV = 65–75 mm; slice thickness = 0.6 mm; Kernel = U90 *μ*. Each MDCT image has a size of 512 × 512 × 58 voxels with 0.12x0.12x1.0 mm spacing.

Preoperative and postoperative CBCT images were done on a Morita 3D Accuitomo 170 (J. Morita). Each ear was imaged separately using the following parameters: 90-kV tube voltage; 5-mA current; a high-resolution mode (Hi-Res J.Morita) with a rotation of 180 degrees; voxel size of 0.125 mm and FOV of 80x80x80 mm. The final postoperative CBCT images have a size of 485 × 485 × 121 voxels with 0.12 × 0.12 × 0.5mm spacing and the final preoperative CBCT images have a size of 483 × 483 × 161 voxels with 0.12 × 0.12 × 0.3 mm spacing. These CBCT images were probably cropped as they don’t have isotropic spacing.

### ACIR: Automatic Cochlea Image Registration

The multi-registration Automatic Cochlea Image Registration (ACIR-v1) was proposed in [13] to provide practical cochlea image registration in a few seconds. It combined different standard techniques and tuned parameters in a customized way that worked for cochlea images. It combined different registration elements in a hierarchical approach of two stages. In the first stage, a rigid registration was used while in the second stage, a non-rigid B-*spline* registration was used. Moreover, the metric was based on the input image types i.e *S*_*MSE*_ for monomodal images i.e CBCT and CBCT, and *S*_*MMI*_ for multimodal images e.g. MDCT and MR, or MDCT and CBCT. Even though CBCT and MDCT are very similar, in practice, CBCT and CT had better image registration result when *S*_*MMI*_ was used. It used the Adaptive Stochastic Gradient Descent (ASGD) as an optimiser [14]. The step-size λ, in Eqs (3) and (5), was an important optimisation factor that had to be set manually. It had a large influence on the GD optimisation results. This factor is data-dependent, so finding a suitable value for different problems was challenging. ASGD is an optimiser that adapts the step-size factor λ automatically, using an image-driven mechanism, to predict its value. It replaces λ with a parameter *δ* that defines the maximum incremental displacement allowed between the optimisation’s iterations. The *δ* parameter was computed using the voxel size of the input image. The mean voxel spacing (in mm) was found to be a good value.

The cochlear image registration problem in this study is an intra-subject image registration problem. Hence, it belongs to rigid image registration problems. For such images, the same transform applies to all pixels in the moving image. Finding the correct transform for any part of the moving image may solve the problem for other parts in the image. Some areas in these images have clear structures and less noise. Cropping the original images to one of these small areas and registering them is an efficient way to produce a transform that registers the original images. Another method, ACIR-v2, which uses prior knowledge of the images’ characteristics was introduced in [15]. ACIR-v2 uses the ASGD optimiser to minimise negative Mattes’ mutual information *S*_*MMI*_ similarity metric of two images [16] by modifying 3D rigid transformation’s parameters.

ACIR-v1 and ACIR-v2 were GD methods. In GD, the second order derivative of the similarity metric function, also called the Hessian *H*, was the identity matrix *I*, hence, it was omitted from (3). In some cases where the Hessian matrix is ill-conditioned, this assumption leads to an inefficient optimisation result. In this case, one should use a Newton optimiser method where the second-order partial derivatives of the cost function are considered. This approach is very computationally expensive as the computation of the Hessian requires a lot of time and memory capacity.

### The proposed idea

Quasi-Newton methods [7] use an approximation of the Hessian of the cost function which reduces the computation time. The update function in quasi-Newton optimiser is:

$$\begin{array}{c} \mu_{k+1}=\mu_{k}-\lambda (\frac{\left(\frac{\partial S_{k}\left(I_{F}\left(P\right),I_{M}\left(T\left(P,\mu_{k}\right)\right)\right)}{\partial \mu }\right)}{\left(\frac{\partial^{2}S_{k}\left(I_{F}\left(P\right),I_{M}\left(T\left(P,\mu_{k}\right)\right)\right)}{\partial \mu^{2}}\right)}), \end{array}$$ (6)

where *k* is the current optimisation iteration, $\frac{\partial S_{k}}{\partial \mu }$ is the first order derivative of the similarity metric function at iteration *k*, λ is the step-size and $\frac{\partial^{2}S_{k}}{\partial \mu^{2}}$ is a symmetric positive definite approximation of the Hessian matrix. A popular quasi-Newton update rule is Broyden-Fletcher-Goldfarb-Shanno (BFGS) [7] which uses the first-order derivatives to update the inverse Hessian directly. This produces a linear rate convergence. The BFGS update rule is described in Eq (7):

$$\begin{array}{c} H_{k+1}=V_{k}^{T}H_{k}^{-1}V_{k}+\frac{\mu_{k}^{\prime }{\mu^{\prime }}_{k}^{T}}{{g^{\prime }}_{k}^{T}\mu_{k}^{\prime }}\;, \end{array}$$ (7)

where $V_{k}=I-\frac{g_{k}^{\prime }{\mu_{k}^{\prime }}^{T}}{{g_{k}^{\prime }}^{T}{\mu^{\prime }}_{k}}$, *I* is the identity matrix, $g_{k}^{\prime }=\frac{\partial S_{k+1}}{\partial \mu }-\frac{\partial S_{k}}{\partial \mu }$, $\mu_{k}^{\prime }=\mu_{k+1}-\mu_{k}$, $H_{k}^{-1}$ is the inverse matrix of $\frac{\partial^{2}S_{k}}{\partial \mu^{2}}$, *V*^*T*^ is the transpose of *V*, and *μ*^*T*^ is the transpose of *μ*.

This still requires a long computation time and large memory. The memory issue is solved by the Limited-memory BFGS (LBFGS) [17] method which saves only a few numbers of the previous Hessian approximations. The computation time issue is solved using a stochastic approach.

This paper introduces the Automatic Cochlea Image Registration (ACIR-v3) method which is based on the stochastic quasi-Newton with Limited-memory BFGS (s-LBFGS) updating rule.

The proposed method replaces the ASGD optimiser in ACIR-v2 with the s-LBFGS optimiser [18]. The s-LBFGS combines the stochastic approach with the LBFGS approach. This allows for faster convergence and more robust results.

### Evaluation method and statistical analysis

The ACIR-v3 method was compared to its previous versions (ACIR-v1, ACIR-v2), and three other optimisers i.e. ASGD, Fast Adaptive Stochastic Gradient Descent (FASGD) [19], and Fast Preconditioner Stochastic Gradient Descent (FPSGD) [20]. Since the result time recorded might differ according to the hardware used, all the experiments were done using the same hardware. The hardware used was a computer equipped with AMD Ryzen 3900 CPU, a 32 GB memory and a Nvidia RTX2080Ti graphics card. We used the same parameters for all methods and the original implementations of ACIR-v1, ACIR-v2, ASGD, s-LBFGS, FPSGD by their authors which are provided as an open-source in 3D Slicer software and Elastix 5.0.0 toolbox [21, 22].

In the ASGD method, we used the original images and no cropping was involved. The ASGD parameters were: rigid transformation, no multi-stages, and no multi-resolution. The optimiser was changed in order to make fair comparisons between FASGD, FPSGD, ACIR-v2, and ACIR-v3. The comparison between the methods included three criteria: the image registration accuracy, the required time to align a pair of images, and the robustness of the method. The time was recorded using a fixed number of iterations (n = 100) and the pre-processing and post-processing steps, e.g. image cropping, were included. The robustness of the method was evaluated using the percentage of the missing results according to Eq (8):

$$\begin{array}{c} \frac{N_{success}}{N_{success}+N_{fail}}\;, \end{array}$$ (8)

where *N*_*success*_ is the number of cases where the method produced a valid result, and *N*_*fail*_ is the number of cases where the method failed to produce a valid result e.g. the optimisation stopped.

**Algorithm 1**: ACIR-v3

1 **Input**: two cochlea images *I*_*F*_(*P*), and *I*_*M*_(*P*);

2 **Output**: a registered and fused image *I*_*result*_(*P*);

3 Locate the cochlea locations in input images;

4 ${\tilde{I}}_{F}$, ${\tilde{I}}_{M}=\text{Crop}(I_{F},I_{M})$;

5 Set transform *T* = 3D rigid transform;

6 Set *k* = 0.;

7 **While** $-S_{MMI}({\tilde{I}}_{F}\left(P\right),{\tilde{I}}_{M}\left(T\left(P,\mu \right)\right)$ is large and *k* < 100 **do**;

8  *μ* = update the old *μ* using s-LBFGS.;

9  Set *k* = *k* + 1;

10 Transform the moving image, *I*_*result*_(*P*) = *I*_*M*_(*T*(*P*, *μ*));

11 Fuse the result, *I*_*result*_ = *I*_*result*_ + *I*_*F*_

**Fig 3:**
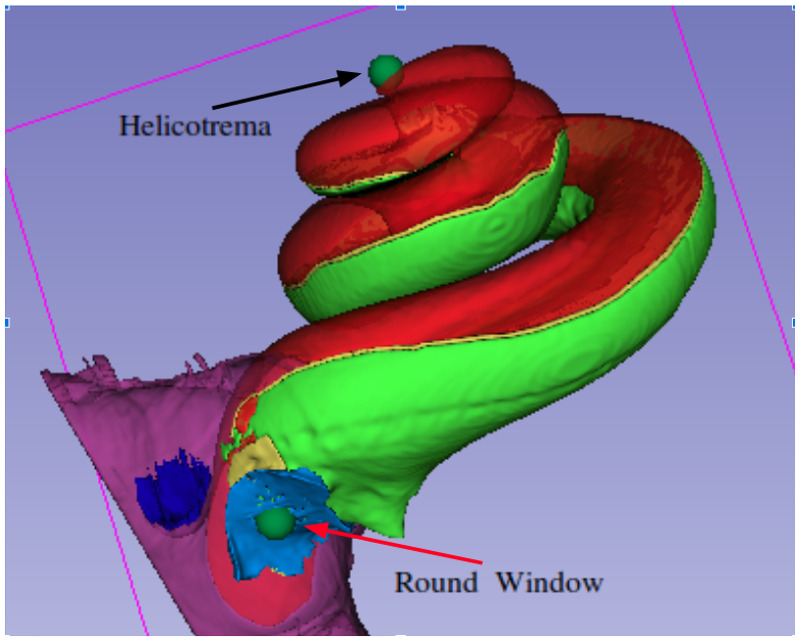
Cochlea 3D models and two landmarks. This model is generated in our lab by manual segmentation of a high quality *μ*CT image using 3D Slicer software. The red colour represent scala media and scala vestibuli. The green colour represent the scala tympani.

Two neuroradiology experts added two 3D landmark points (fiducial points) (in consensus) to all the imaging studies using the 3D Slicer software version 4.10 [23, 24]. The two landmark points represent the round window and the cochlear apex, see Fig 3. These landmarks were saved for each imaging study and were used later for validation of the image registration results. After aligning the images, the landmarks of the moving image were transformed using the same transformation. Thereafter, RMSE in mm was measured between these transformed landmarks and the related fixed image landmarks.

For justification, the results were repeated 3 times and the average values were used. The results have been divided into 4 groups based on image modalities to obtain more details on how each method performs on a specific modality. This allowed for more detailed evaluation as some methods may be biased to specific types of images. These groups were (CBCT, CBCT), (CBCT, MR), (CBCT, MDCT), and (MR, MDCT). In addition to these group results, the total results were presented to give a global evaluation of each method. The average RMSE and average time calculated for each registration process in each of the 6 methods were then used to compare (ACIR-v3) to each of the other 5 methods using independent T-tests.

### Other optimisers (used for comparisons)

FASGD is another adaptive stochastic optimiser that estimates the step-size automatically using the observed voxel displacement. During the computation of the step-size, two ASGD parameters are fixed in FASGD, while they have to be computed in each iteration in ASGD. This makes FASGD faster but less adaptive than ASGD.

Preconditioned Stochastic Gradient Descent (PSGD) [25] is proposed to improve the rate of convergence of GD methods. It adds a preconditioning strategy to Robbins-Monro stochastic gradient descent (SGD_*RM*_) [11] and ASGD methods. The updating rule for PSGD is:

$$\begin{array}{c} \mu_{k+1}=\mu_{k}-\lambda M\frac{\partial S_{k}\left({\hat{I}}_{F}\left(P\right),{\hat{I}}_{M}\left(T\left(P,\mu_{k}\right)\right)\right)}{\partial \mu }\;. \end{array}$$ (9)

The equation is similar to Eq (5), the only difference is adding the preconditioner matrix *M* which is a symmetric positive definite matrix with size equal to the number of the transformation’s parameters, i.e. |*μ*|. When *M* is the identity matrix, we get the standard SGD method as in Eq (5). In [25] the step-size is defined to be a non-increasing and non-zero sequence to guarantee convergence:

$$\begin{array}{c} \lambda_{k}=\{{\begin{array}{cc} 1 & \text{if}\;k=0\\ \frac{\eta }{(\left(t_{k}+1\right)/A)+1} & \text{if}\;k>0 \end{array}}_{,} \end{array}$$ (10)

where *t*_0_ = *t*_1_ = 0, $t_{k}=max(0,t_{k-1}+sigmoid\left({\tilde{g}}_{k-1}M{\tilde{g}}_{k-2}\right)$, $\tilde{g}$ is an approximation of $\frac{\partial S_{k}}{\partial \mu }$, *M* is the preconditioner matrix, *A* = 20 is a decay speed factor, and *η* is a noise factor and is defined as:

$$\begin{array}{c} \eta =\frac{E||g^{T}Mg\left|\right|}{E||{\tilde{g}}^{T}M\tilde{g}\left|\right|}=\frac{E||g^{T}Mg\left|\right|}{E||g^{T}Mg||+E||\epsilon^{T}M\epsilon \left|\right|}\;, \end{array}$$ (11)

where *g* is the exact gradient and *ϵ* is a random noise added to the exact gradient. Unfortunately, PSGD is limited to monomodal image registration where the fixed and the moving images are the same type e.g. MDCT. The FPSGD solved this issue and can be used for multimodal image registration. The authors mention that FPSGD is 2 to 5 times faster than SGD methods while retaining the same accuracy [20].

FPSGD estimates the diagonal entries of a preconditioning matrix *M* on the distribution of voxel displacements. This rescales the registration similarity metric and allows for more efficient optimisation. Let *M* be a preconditioning matrix with the same length of the parameter vector *μ*, and *m*_*i*_ is an element in the diagonal of *M* that can be computed using Eq (12):

$$\begin{array}{c} m_{i}=\frac{\delta }{E(||J_{\mu_{i}}\left||.|\right|\tilde{g_{i}}\left||\right)+2\sqrt{Var(||J_{\mu_{i}})||.||\tilde{g_{i}}\left||\right)}+\epsilon }\;, \end{array}$$ (12)

where *δ* is a pre-defined value representing the maximum voxel displacement, *E*||*X*|| is the expectation of the *l*_2_ norm, *Var*||*X*|| is the variance of the *l*_2_ norm, $J_{\mu_{i}}$ is the i_*th*_ column in the Jacobian matrix $J_{\mu }=\frac{\partial T(P,\mu )}{\partial \mu }$ which is mentioned earlier in Eq (4), and *ϵ* is a small number to avoid dividing by zero.

## Results

Out of the 131 imaging studies included in the experiments, 124 registration processes (CBCT, CBCT), (CBCT, MR), (CBCT, MDCT), and (MR, MDCT) were performed using the 6 included methods (ASGD2009, FASGD2015, FPSGD2019, ACIR-v1, ACIR-v2, and ACIR-v3). The mean RMSE, the mean time used for each method, and the results of their comparison to ACIR-v3 using the independent samples T-test are shown in Tables 1 and 2. N represents the number of registration processes, and SD represents the standard deviation. The registration processes failed in FASGD2019 in 2/124 and in FPSGD2019 in 13/124.

**Table 1:** Independent samples T-test for the RMSE of each method in comparison to ACIR-v3.

Method	N	RMSE (mm)	SD	*p*-value
ASGD2019	124	14.98	8.63	<0.0001
FASGD2015	122	2.77	11.85	0.03
FPSGD2019	111	1.45	8.39	0.194
ACIR-v1	124	10.95	9.497	<0.0001
ACIR-v2	124	0.36	0.17	0.125
ACIR-v3	124	0.41	0.30	-

N: number of registration experiments. RMSE: root mean squared error. SD: standard deviation. *p*-value: statistical samples t-test *p*-value comparison to ACIR-v3.

**Table 2:** Independent samples T-test for the time of each method in comparison to ACIR-v3.

Method	N	Time (seconds)	SD	*p*-value
ASGD2019	124	6.45	0.83	<0.0001
FASGD2015	122	4.76	1.23	0.37
FPSGD2019	111	4.70	1.19	0.601
ACIR-v1	124	15.97	3.37	<0.0001
ACIR-v2	124	4.65	1.2	0.86
ACIR-v3	124	4.62	1.2	-

N: number of registration experiments. SD: standard deviation. *p*-value: statistical samples t-test *p*-value comparison to ACIR-v3.

**Fig 4:**
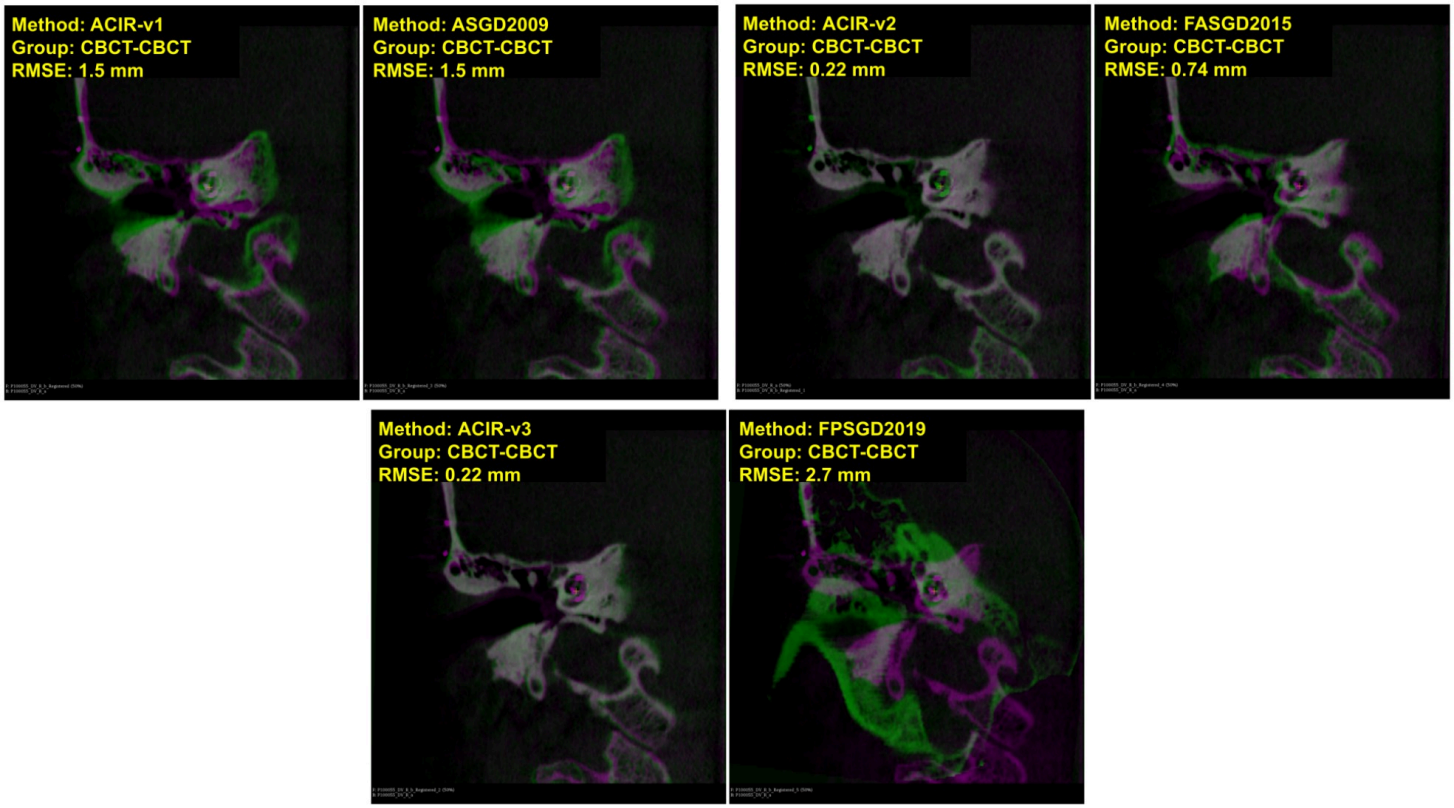
Sample results of registration CBCT to CBCT (coronal views). The figure shows image types, methods, and RMSE information. Magenta represents the fixed image and green represents the moving image.

**Fig 5:**
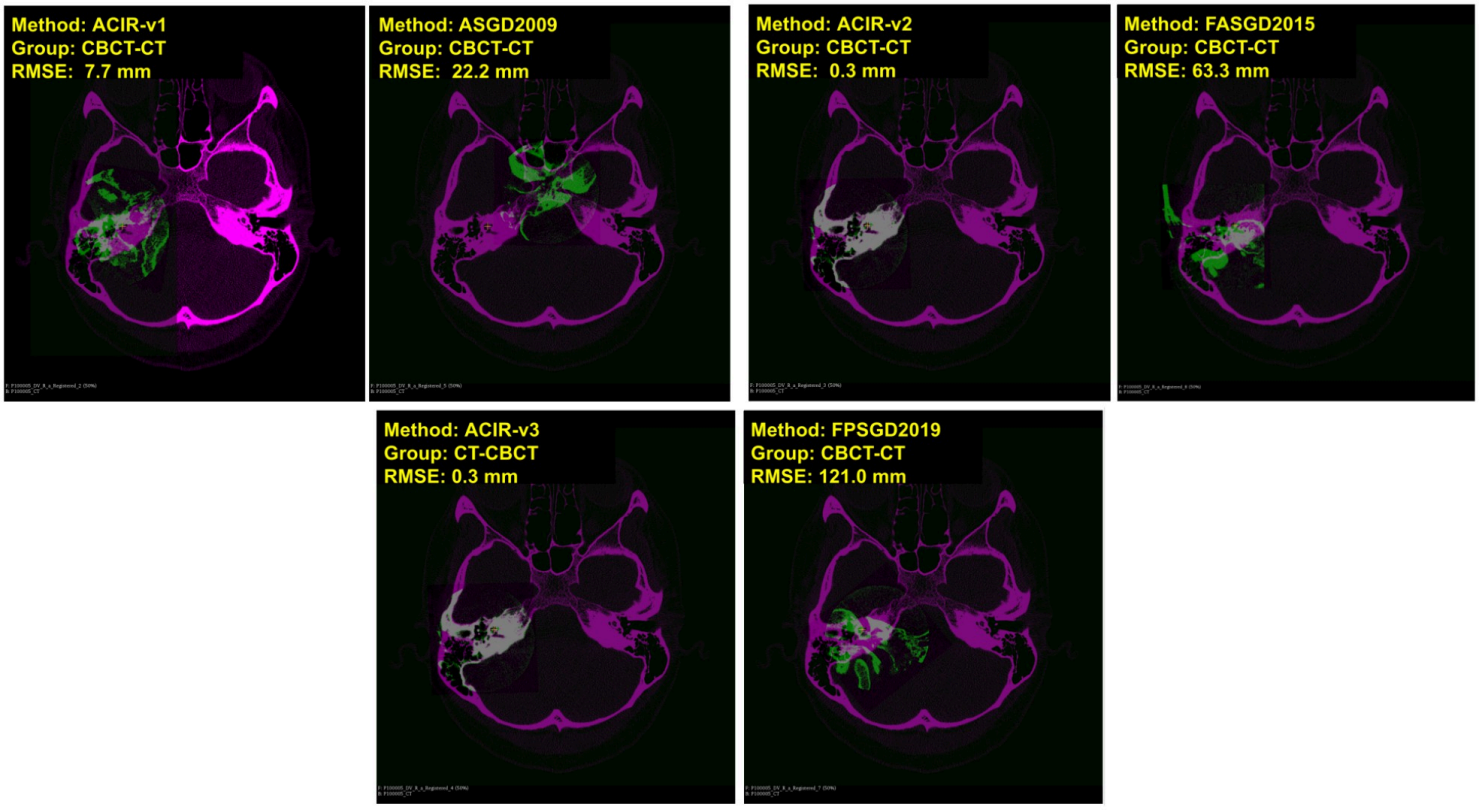
Sample results of registration CBCT to MDCT (axial views). The figure shows image types, methods, and RMSE information. Magenta represents the fixed image and green represents the moving image.

**Fig 6:**
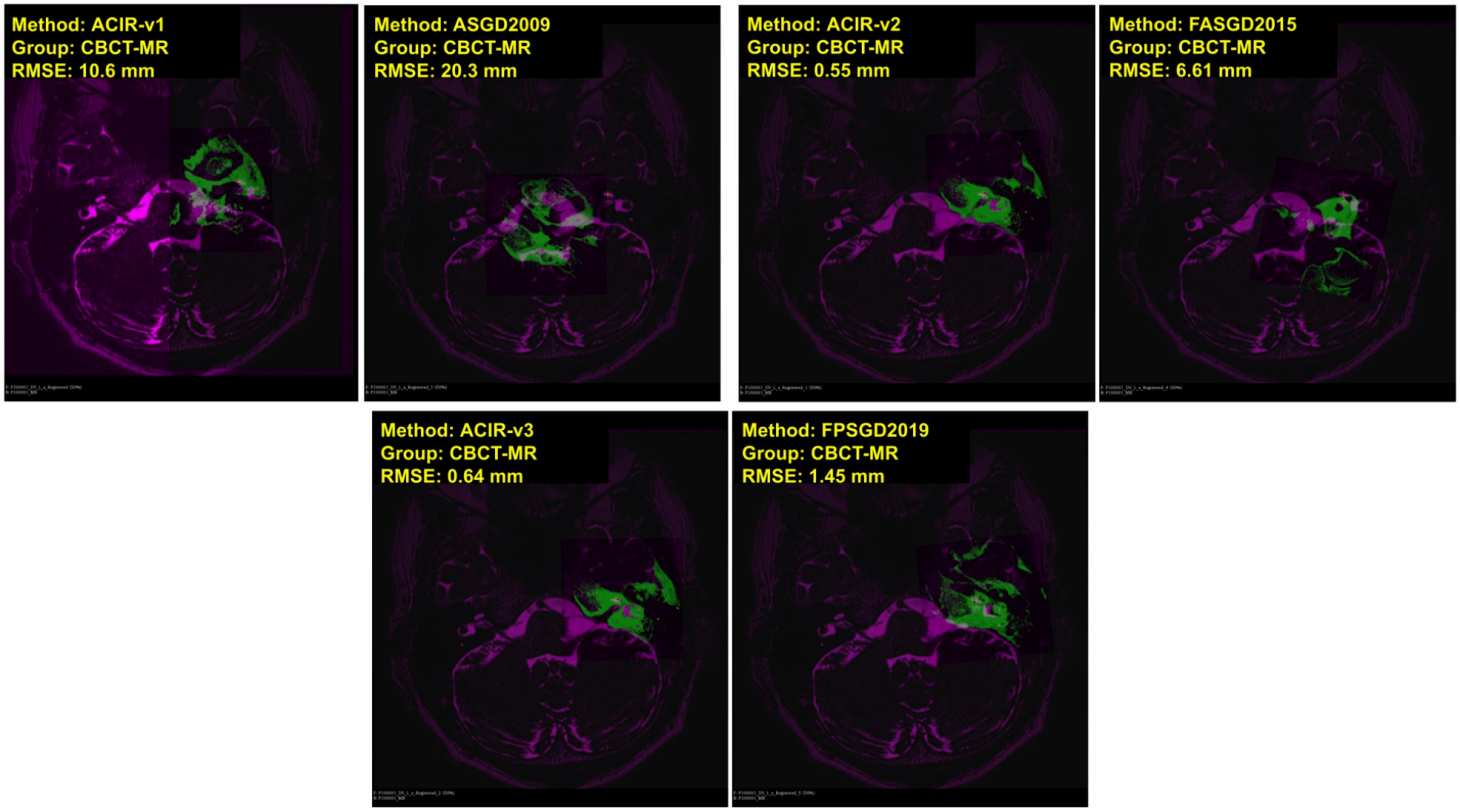
Sample results of registration CBCT to MR. The figure shows the axial views with image types, methods, and RMSE information. Magenta represents the fixed image and green represents the moving image.

**Fig 7:**
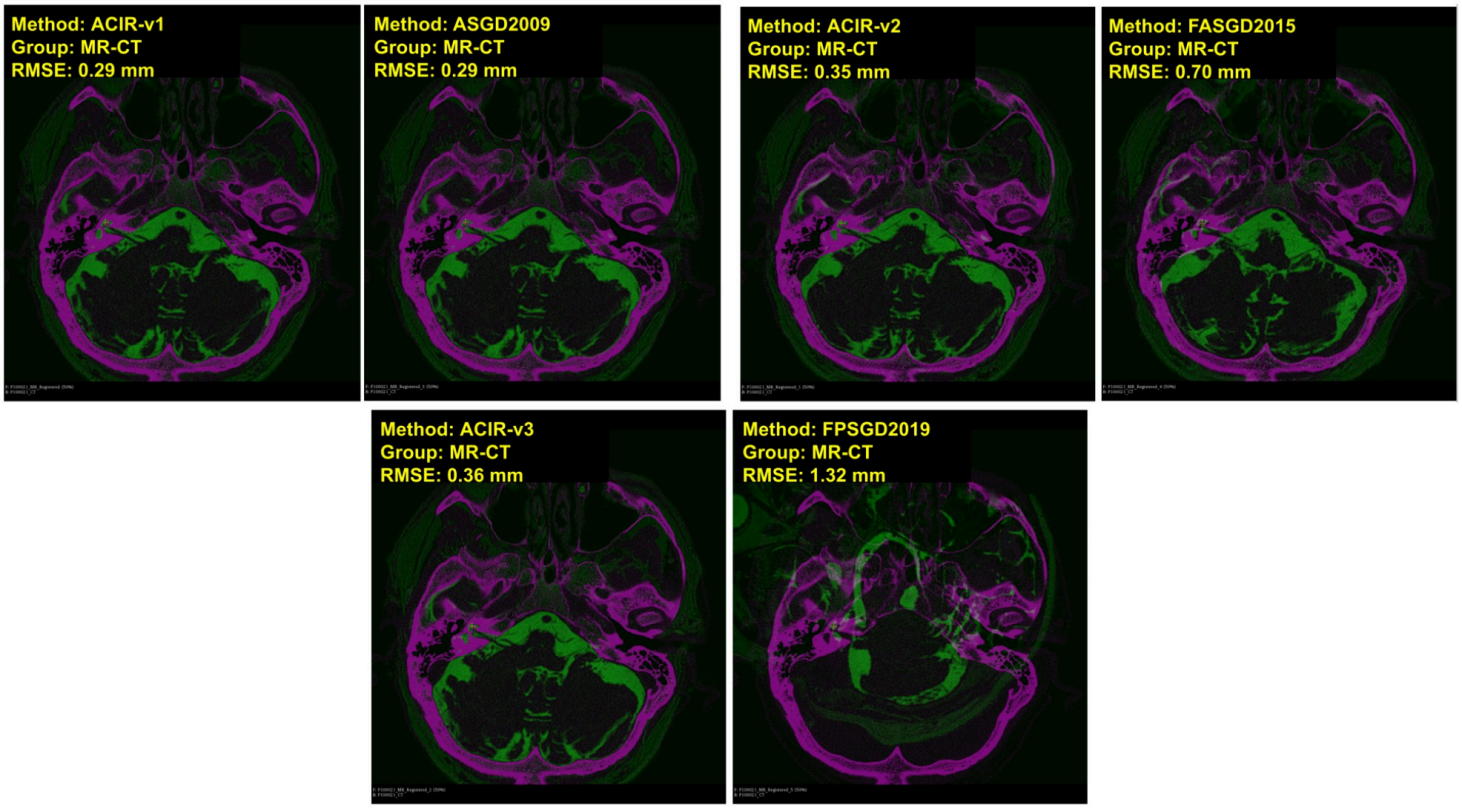
Sample results of registration MR to MDCT. The figure shows the axial views with image types, methods, and RMSE information. Magenta represents the fixed image and green represents the moving image.

In Figs 4 and 5, visual samples of CBCT to CBCT, and CBCT to MDCT image results of all 6 methods are shown. The fixed image is in magenta and the moving image is in green. The distance between the ground truth and the registered ground truth landmarks is also shown. When more green and magenta can be seen, the registration process is inadequate as seen in the bottom right image of Fig 4 where FPSGD2019 method is used. When the green and magenta are mixed, the registration process is successful as seen in the bottom left of Fig 4 where ACIR-v3 is used. Similarly, Figs 6 and 7 show visual samples of CBCT to MR, and MR to MDCT image registration and fusion results. We selected different modalities and different views to give a visual sample of the results.

**Fig 8:**
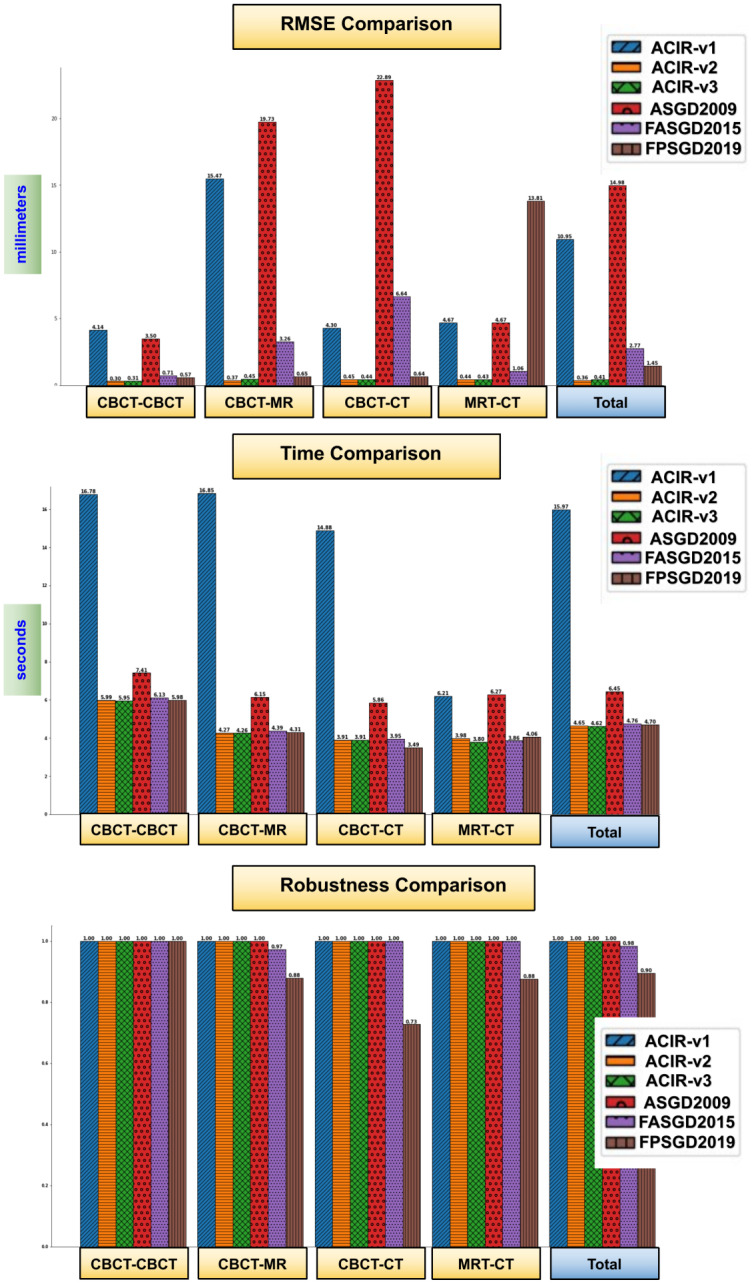
Results chart, the average RMSE, time, and robustness of each tested method grouped based on image-modalities. The robustness is computed based on Eq (8).

The three charts in Fig 8 show a comparison of the 6 methods in terms of accuracy, time, and robustness. ASGD, ACIR-v2, and ACIR-v3 methods were 100% robust.

## Discussion

There are few scientific papers related to cochlea image registration. Manual image registration and fusion are usually done by doctors which requires much effort and time as described in [26]. The authors proposed a manual procedure for image registration and fusion of CT and MR of the temporal bone using bony surgical landmarks. This procedure took 13 minutes per scan.

In [27], an automatic cochlea image registration for CT was proposed for Percutaneous Cochlear Implantation (PCI) surgery. The authors reported a maximum of 0.19 mm error when using their method. The method was complex as it involved using segmentation and two-stage registration. The computation time required to complete the registration was approximately 21 minutes which was larger than the time required for manual registration (approximately 10 minutes based on our experiments). The authors investigated only complete head CT scans so the functionality of their method with multi-modal images or small images focused on the cochlea aspect is not clear.

In the other research, high-resolution *μ*CT data was used in [28, 29] where two methods were proposed for cochlea image registration. The first method was based on heat distribution similarity in a cubic B-*splines* registration model. The second method was based on skeleton similarity as an anatomical prior. Their methods required cropped and segmented images which was a time consuming process that took approximately 48 minutes per scan.

Lately, a proposed cochlea registration method was used in [30]. The fusion of sequential CBCT was compared to the gold standard fiducial in order to analyse clinical CI migration. BRAINSFit tool in 3D Slicer was used which reduced the duration of the registration to less than 2 minutes. A mean error of 0.16 mm was reported. However, this method did not support multimodal images, and the images used did not include cochlea implants which made the registration problem less challenging.

Automatic Cochlea Image Registration and fusion (ACIR-v1) was the first attempt to achieve practical automatic cochlea image registration. The method’s robustness was very high, only one case lacked results, but some images required a manual correction step which took a few seconds. The average RMSE error was less than 5 mm for all image types except (CBCT, MR) where it was 15.47mm. It seems that for these two image types, the noise level was too high which made the mutual information unclear and so it could not be maximised. The time required to process a pair of images was high because of the use of the B-*spline* transform which has thousands of parameters. Compared to ASGD, ACIR-v1 produced a 3.8% improvement in accuracy.

The cropping in ACIR-v1 divided the input images to include the targeted cochlea i.e. 50% of the image represents either the left or the right ear. In ACIR-v2, the cropping was reduced to the cochlea region which was around 10x10x10 mm. Additionally, the multi-resolution and the multi-registration approaches were removed. This produced better accuracy and faster computation. This showed that the cropping approach in ACIR-v2 was instrumental in achieving practical results and using ASGD alone was not enough.

In this study, the ACIR-v3 method has been proposed based on the quasi-Newton s-LBFGS method. The history of the method’s development was outlined i.e. ACIR-v1, and ACIR-v2. The method presents a practical and fast solution for the multimodal cochlea 3D image registration and fusion problem. The method results have been validated using landmarks located by two experts and tested using 131 multimodal cochlea 3D images of 41 patients from CBCT, MDCT, and MR modalities. The method aligned all the images successfully in a few seconds. The results showed that using ASGD or s-LBFGS has higher accuracy than FASGD or FPSGD optimisers, especially concerning multimodal images. This is due to the FASGD method being less adaptive than ASGD, whilst the FPSGD method has higher sensitivity to noise. In low-resolution images with complicated structures, such as clinical images of the cochlea, more noise is included which leads to poor alignment when FPSGD is used. ACIR-v3 has 4.8% accuracy and 0.05% speed enhancement over the FPSGD method on average.

The comparison of the ACIR-v3 to its previous versions (ACIR-v1 and ACIR-v2), showed a statistically significant difference between ACIR-v3 and ACIR-v1 with much improvement in the time used and lower RMSE. However, there was no statistically significant difference between ACIR-v3 and ACIR-v2.

The comparisons of ACIR-v3 with the known methods showed no statistically significant differences in terms of the mean time used except for (ASGD2009), where ACIR-v3 was relatively faster (*p* < 0.0001). The comparison of the RMSE in ACIR-v3 to ASGD2009 (*p* < 0.0001) and FASGD2015 (*p* = 0.03) was statistically significant. The comparison of the RMSE of the FPSGD2019 to ACIR-v3 showed no statistical significance, yet the mean RMSE was relatively higher in FPSGD2019 (1.45±8.4 mm compared to only 0.41±0.3 mm in ACIR-v3). However, the registration process failed in 11 out of the 124 processes in FPSGD2019 which demonstrated instability.

### Limitation

The image registration field is very rich. There are many methods published every year and one cannot cover all the related work in one paper. Hence we selected related work close to our problem which involves rigid transformation and cochlea images. However there is some interesting work which can be used for problems involving non-rigid image registration and different datasets as in [31–35]. Deep learning methods are increasing in popularity in medical image registration e.g. as in [36, 37]. However, they require expensive specialized hardware and thousands of medical images for training which are often unavailable. Moreover, the accuracy of these methods is poor in some tasks [38].

## Conclusions

The proposed method’s source-code is provided as a public open-source and can be downloaded from a public server. The raw cochlea medical image dataset cannot be made publicly available at present for legal reasons. However, all the original results tables are publicly available. This enables other researchers to reproduce these experiments and for people to benefit from the results.

Future work will include studying more s-LBFGS parameters and testing the method against different problems and on different datasets.

## Supporting information

S1 Data (CSV)
